# Serum and Ectopic Endometrium from Women with Endometriosis Modulate Macrophage M1/M2 Polarization via the Smad2/Smad3 Pathway

**DOI:** 10.1155/2018/6285813

**Published:** 2018-09-12

**Authors:** Mei-Fang Nie, Qi Xie, Ya-Hong Wu, Hua He, Lu-Jie Zou, Xiao-Ling She, Xian-Qing Wu

**Affiliations:** ^1^Department of Obstetrics and Gynecology, The Second Xiangya Hospital of Central South University, Changsha, Hunan Province 4100011, China; ^2^Department of Pathology, The Second Xiangya Hospital of Central South University, Changsha, Hunan Province 410011, China

## Abstract

**Objective:**

This study investigated the alterations in macrophage polarization in patients with endometriosis as well as the underlying molecular mechanisms.

**Methods:**

Peritoneal washings, serum samples, and endometrial tissues were collected from endometriosis patients and control subjects. Endometrial stromal cells (ESCs) were isolated from endometrial tissue, and conditioned medium was prepared by treating ESCs with or without various concentrations of interleukin- (IL-) 6, estrogen, or progestin. The frequencies of CD86+ and CD163+ cells and expression levels of these markers as well as the cytokines IL-12 and IL-10 were measured in THP-1- (human monocytic leukemia cell) derived macrophages.

**Results:**

There was a decrease in the percentage of CD86+ macrophages in the peritoneal wash solution of patients with endometriosis. Ectopic endometrial homogenates could promote M1 to M2 macrophage polarization in response to lipopolysaccharide (LPS), as evidenced by the increased percentage of CD163+ macrophages and increased IL-10 expression as well as a decreased percentage of CD86+ cells and lower IL-12 expression. In contrast, addition of serum from women with endometriosis to THP-1 cells resulted in the polarization of macrophages towards both M1 and M2 phenotypes. Upregulation of Smad2/Smad3 in macrophages upon exposure to eutopic and ectopic endometrial homogenates as well as serum of women with endometriosis was observed, and blockage of Smad2/Smad3 with their inhibitor SB431542 could reverse the macrophage polarization from M1 to M2. Conditioned medium induced by IL-6, but neither estrogen nor progestin, could facilitate M2 polarization. Neutralization of IL-6 diminished macrophage M2 polarization in endometriosis.

**Conclusion:**

This study provides detailed evidence supporting alterations in M1 to M2 macrophage polarization that may contribute to the initiation as well as progression of endometriosis.

## 1. Introduction

Endometriosis is a common nonmalignant gynecological disorder affecting 10–15% of women of reproductive age that manifests with the presence of ectopic endometrial cells and stroma in various locations outside of the uterine cavity, mainly in the peritoneal cavity [1]. Endometriosis may cause abdominal pain, dysmenorrhea, dyspareunia, and especially infertility, leading to profound physical and psychological distress [2]. A convincing hypothesis for the etiology of endometriosis has been that endometrial debris is refluxed by retrograde menstruation implant into the abdominal cavity where it grows and induces chronic inflammation with formation of adhesions. During this process, alterations in multiple aspects of humoral and cell-mediated immunity contribute to the pathogenesis of endometriosis [3–5]. Decreases in T cells and natural killer cells and modulation of peritoneal macrophages result in inadequate removal of ectopic endometrium from the peritoneal cavity. Furthermore, local and systemic immune factors, cytokines, and growth factors that are secreted by either immune or endometrial cells may favor the ectopic implantation and growth of endometrial cells. Susceptibility factors such as genetic predisposition, environmental factors, and immunodeficiency make it easier for endometriotic tissue to implant and survive in the peritoneal environment [6, 7].

Macrophages are phagocytic cells of the immune system that distribute in various tissues and play a critical role in various diseases such as inflammatory disorders and the growth of tumors. Based on their roles, macrophages are broadly classified into M1 macrophages (known as classically activated macrophages) and M2 macrophages (known as alternatively activated macrophages). M1 macrophages, which express specific biomarkers CD40, CD80, CD86, and human leukocyte antigen-antigen D related (HLA-DR), are potent effector cells that eliminate invading microorganisms and secrete proinflammatory cytokines, such as interleukin- (IL-) 1*β*, IL-6, IL-12, and tumor necrosis factor-*α* (TNF-*α*). In contrast, M2 macrophages, which express specific markers CD163 and CD206, ameliorate inflammation and produce many anti-inflammatory factors such as IL-10, TGF-*β*, and IL-1 receptor antagonist (IL-1ra), but very low levels of proinflammatory cytokines such as IL-12 [8]. Macrophages are a group of heterogeneous and plastic cells that switch from M1 to M2 phenotype, and vice versa, upon the induction of specific signals. M1 macrophages can be directly driven to the M2 phenotype by canonical M2 stimuli like exposure to IL-4 and IL-10 [9]. Moreover, several signal pathways have been implicated in the M1/M2 polarization of macrophages [10, 11]. For example, the Toll-like receptor (TLR)/nuclear factor kappa B (NF-*κ*B) signaling pathway reprograms macrophages to the M1 phenotype in response to microbial invasion [10], whereas TGF-*β* can switch macrophages to the M2 phenotype via the SMAD2/3/4-dependent pathway [12].

Endometriosis is a chronic inflammatory disorder, and symptomatic cases with peritoneal lesions have been associated with dysregulated cytokine production and differential expression of immune-inflammation genes in both ectopic and eutopic endometrium, accompanied by elevated bacterial load and endotoxin level in the peritoneal environment [13–15]. Under such conditions, the disruption of the dynamic balance between M1 and M2 macrophage phenotypes may contribute to the pathogenesis of endometriosis. It has been reported in a mouse model that endogenous macrophages are involved in tissue remodeling during the development of endometriosis, and M1 to M2 phenotypic transition is required for the growth of ectopic lesion [16]. However, the mechanisms by which alterations in macrophages induce and safeguard endometriotic lesions at ectopic sites in patients with endometriosis remain poorly understood. In this study, we measured the polarization status of macrophages in the peritoneal fluid of patients with endometriosis. The phenotypic switch of macrophages was also observed when THP-1- (human monocytic leukemia cell) derived macrophages were subjected to eutopic and ectopic endometrial homogenates as well as serum from endometriosis patients, and the underlying signaling pathways were explored.

## 2. Methods and Materials

### 2.1. Endometrial Tissue

The experiment was approved by the Ethics Committee of Second Xiangya Hospital of Central South University. All patients signed an informed consent form prior to participation. A flow chart of the study is presented in Supplementary Figure 1. The endometrial tissue samples were collected during laparoscopic surgery from 25 patients with a histologically confirmed diagnosis of endometriosis. These patients were admitted to our hospital between December 2015 to December 2016 due to an ovarian cyst or infertility. In accordance with the revised American Society for Reproductive Medicine (rASRM) classification, 6 patients had stage I-II endometriosis, 5 patients had stage III, and 14 patients had stage IV. The surgery was performed in either the proliferative (*n* = 20) or secretory (*n* = 5) phase of the menstrual cycle. Another 12 patients who underwent laparoscopic surgery for incision of the uterine septum during the same period were enrolled, among which 8 cases were in the proliferative phase and 4 cases were in the secretory phase. The absence of endometriosis in these patients was confirmed laparoscopically. All participants were aged 25–40 years old and had a regular menstrual cycle. No patients had received hormone therapy within 3 months before laparoscopic surgery.

The abdominal cavity was washed with 50 ml normal saline, and peritoneal washings were collected into a centrifuge tube. Under aseptic conditions, the endometrium and ectopic lesions (chocolate cyst wall) were scraped. The specimens were rinsed with sterile phosphate-buffered saline (PBS) three times, then immediately preserved in sterile PBS supplemented with 1% penicillin and streptomycin, and transferred to the laboratory. The tissues were fixed in 10% paraformaldehyde or stored in liquid nitrogen for further use.

### 2.2. Mononuclear Cell Isolation in Peritoneal Fluid

The peritoneal washings collected were centrifuged at 1000 rpm for 5 min. The cell pellet was harvested and resuspended in 4 ml PBS. The mononuclear cells were isolated by density gradient centrifugation with Ficoll-Paque (GE Healthcare Biosciences, Pittsburgh, PA, USA). Briefly, Ficoll-laid samples were centrifuged at 2200 rpm for 20 min. The buffy layers containing cells were carefully transferred to new tubes for resuspension and washed in PBS twice at 1200 rpm for 10 min to obtain mononuclear cells.

### 2.3. Preparation of Endometrial Homogenates

Endometrial tissue homogenates were prepared as previously described [17]. Each of 10 frozen endometrial tissue samples (200 mg) was immediately placed in 5 ml of serum-free RPMI 1640 medium and homogenized in a glass homogenizer. The sample mixture was centrifuged at 1200 rpm for 10 min. The supernatant was then collected and stored frozen at −80°C for further use.

### 2.4. Collection of Serum Samples

A 5-ml fasting venous blood sample was collected from each patient on the morning of surgery and placed in a tube with procoagulants. The samples were centrifuged at 1200 r/min for 8 min. The serum samples were frozen at −80°C for further use.

### 2.5. Isolation and Culture of Endometrial Stromal Cells (ESCs)

The endometrial tissue was minced to pieces of about 1 mm^3^, digested in 4-5 ml of a 0.1% collagenase I solution, and placed in an incubator at 37°C. The mixture was centrifuged at 1000 rpm for 5 min, and the pellet was suspended in 1 ml of serum-free Dulbecco's Modified Eagle Medium (DMEM). The cells were counted, seeded in DMEM medium supplemented with 10% fetal bovine serum (FBS) at a density of 5 × 10^5^/ml, and cultured in a sterile incubator maintained at 37°C with 5% CO_2_.

### 2.6. Preparation of Conditioned Medium

ESCs were cultured in DMEM supplemented with 10% FBS. When the cells reached approximately 80% confluence, the medium was removed and replaced with serum-free medium, with addition of 10^−8^ mol/l estrogen (E_2_), 10^−7^ mol/l progestin (P), 10^−8^ mol/l E_2_ + 10 ng/ml recombinant IL-6 [18], 10^−7^ mol/l P + 10 ng/ml IL-6, 10^−8^ mol/l E_2_ + 10^−7^ mol/l P, or 10^−8^ mol/l E_2_ + 10^−7^ mol/l P + 10 ng/ml IL-6 (PeproTech, Oak Park, CA, USA). The cells were cultured for 24 h, and the supernatant was collected as conditioned medium. For cells treated with IL-6, 2 *μ*g IL-6 neutralizing antibody (Santa Cruz Biotechnology, Santa Cruz, CA, USA) and protein G (Sigma-Aldrich, St. Louis, MO, USA) were sequentially added to the supernatant to neutralize the remaining IL-6 and to precipitate the IL-6/antibody complex, followed by centrifugation at 8000 rpm for 10 min to collect the supernatant.

### 2.7. Culture and Differentiation of THP-1 Cells

The THP-1 cells were a gift from Dr. Huang of the University of South Florida, USA. The THP-1 cells were cultured in RPMI-1640 medium supplemented with 10% FBS, 1% glutamine, 1% penicillin, and 1% streptomycin at 37°C in 5% CO_2_. The medium was changed every 2 days. Upon reaching a high cell density, cells were harvested into a tube and centrifuged at 1000 rpm for 5 min. The pellet was resuspended in RPMI-1640 medium with 10% FBS, and cells were seeded in 6-well plates at a density of 1 × 10^6^ cells/ml. To induce differentiation into macrophages, the cells were stimulated with 25 ng/ml phorbol 12-myristate 13-acetate (PMA; Sigma-Aldrich) for 3 days. The nonadherent cells were removed, and the adherent cells were M0 macrophages.

### 2.8. Cell Treatment Protocol

THP-1 cell-derived macrophages were treated with 100 *μ*l/ml of eutopic or ectopic endometrial homogenate from endometriosis patients or endometrial homogenate from normal participants for 72 h. The cells were then stimulated with 100 ng/ml LPS (Sigma-Aldrich) for 24 h and harvested.

The effects of NF-*κ*Bp50 inhibitor SN50 (MedChemExpress, SML1471, Princeton, NJ, USA) and Smad2/Smad3 inhibitor SB431542 (Selleck Chemical, S1067, Boston, MA, USA) on macrophage polarization were investigated. THP-1 cell-derived macrophages were exposed to 100 ng/ml LPS for 24 h. After resuspension, the cells were treated with 50 *μ*g/ml SN50 for 1 h [19] or 10 *μ*M SB431542 for 24 h [20], followed by addition of 100 *μ*l/ml tissue homogenate for an additional 72 h in coculture. In addition, culture with 20 ng/ml IL-4 (PeproTech, Oak Park, CA, USA) for 5 days served as a positive control.

THP-1 cell-derived macrophages were treated with 100 ng/ml LPS for 24 h, and then cultured in 10% serum from control subjects or endometriosis patients for 6 days, with or without preincubation with 10 *μ*M SB431542 for 24 h. The cells were collected for analysis. In addition, culture with 20 ng/ml IL-4 for 5 days served as positive control.

THP-1 cell-derived macrophages were cultured in DMEM supplemented with conditioned medium in a volume ratio of 2 : 1 for 5 days. The cells were collected for analysis. For neutralizing antibody assay, THP-1 cell-derived macrophages were treated with 100 ng/ml LPS for 24 h and then cultured with 2 *μ*g/ml IL-6 neutralizing antibody or IgG isotype control antibody for another 24 h, followed by incubation with 100 *μ*l/ml eutopic versus ectopic endometrial homogenate for 72 h, or 10% serum from control subjects versus endometriosis patients for 6 days.

### 2.9. Flow Cytometric Analysis

Antibodies against CD86-APC and CD163-PE were purchased from BioLegend (San Diego, CA, USA). Isotypes of these antibodies were used as controls. After blocking with anti-Ig antibody, the cultured THP-1 cells were harvested with trypsin and washed with PBS twice by centrifugation at 2000 rpm for 5 min. The cells were stained with CD86-APC and CD163-PE in the dark at 4°C for 30 min. After the immunological reaction, THP-1 cells were then analyzed on a FACSAria (BD Biosciences, San Jose, CA, USA) using FACSDiva software (BD Biosciences).

### 2.10. Nuclear Protein Extraction

Nuclear protein extraction was performed prior to nuclear NF-*κ*Bp50 determination. The THP-1 cells were scraped off the culture dish and lysed with ice-cold nuclear extraction buffer (1 ml hypotonic buffer containing 5 *μ*l phosphatase inhibitor, 10 *μ*l PMSF, and 1 *μ*l DTT; Sangon Biotech, Shanghai, China). The cells were allowed to swell on ice for 15 min, followed by centrifugation at 3000 rpm for 5 min. The pellet was suspended in hypotonic buffer and then vigorously mixed on a vortex machine at 4°C. The homogenate was centrifuged at 5000 rpm for 5 min. The nuclear pellet was resuspended in lysis buffer, mixed, and incubated on ice for 20 min with intermittent mixing. The mixture was then centrifuged at 15000 rpm for 10 min at 4°C, and the supernatant was nuclear extract, which was stored at −80°C for later analysis.

### 2.11. Western Blotting

The primary antibodies used for western blotting were NF-*κ*Bp50 and *β*-actin (Proteintech, Chicago, IL, USA) and p-Smad2 and p-Smad3 (Abcam, Cambridge, MA, USA). The THP-1 cells were harvested and lysed in 50 *μ*l lysis buffer (Thermo Fisher Scientific, Waltham, MA, USA). The mixture was centrifuged at 14000 rpm for 10 min. Total protein in the supernatant was quantified with a bicinchoninic acid (BCA) kit (Thermo Scientific Pierce, Rockford, IL, USA). Protein samples (30 *μ*g protein) were solubilized and heated at 70°C in NuPAGE® LDS sample buffer and reducing agent (Invitrogen, Carlsbad, CA, USA) for 10 min. Then, 30 *μ*l samples were then loaded onto each lane for electrophoresis and then transferred onto nitrocellulose blotting membranes. The membrane was blocked with 5% fat-free milk at 4°C overnight for 1 h, followed by incubation with primary antibody to Smad2 (dilution 1 : 500), Smad3 (dilution 1 : 200), NF-*κ*Bp50 (dilution 1 : 200), or *β*-actin (dilution 1 : 5000) at 4°C overnight. After washing, the membranes were incubated with goat anti-rabbit secondary antibody (dilution 1 : 4000–6000, Proteintech) at room temperature for 1 h. Immunoreactive bands on the membranes were visualized by enhanced chemiluminescence (ECL; Thermo Pierce, Rockford, IL, USA) detection reagents. The density of each band was quantified by Quantity One software (Bio-Rad Hercules, Hercules, CA, USA) and normalized by reference to the expression value for *β*-actin.

### 2.12. Immunohistochemical Staining

For immunohistochemical staining, the cultured THP-1 cells were fixed in 4% paraformaldehyde for 30 min on slides and washed with PBS. The slides were incubated with 3% hydrogen peroxide for 10 mins to quench endogenous peroxidase activity. After antigen retrieval by EDTA, the slides were blocked with nonimmune serum at room temperature for 1 h, and then incubated with primary antibody against rabbit anti-human CD68 and CD163 (dilution 1 : 500; BioLegend, San Diego, CA, USA) at 4°C overnight. After rinsing with PBS, the slides were incubated with biotinylated goat anti-rabbit secondary antibody (Proteintech) for 30 min. Replacement of the primary antibody with PBS was used for the negative control. The reaction was visualized using 3,3′-diaminobenzidine (DAB; Zhongshan Golden Bridge Biotech, Beijing, China). The slides were rinsed with water and counterstained with Mayer's hematoxylin. The immunological reaction was quantified as the average integrated optical density (IOD) in five random selected fields (400x) using Image-Pro® Plus analysis software (Media Cybernetics, Rockville, MD, USA).

### 2.13. Quantitative Reverse Transcription Polymerase Chain Reaction (RT-PCR)

The total RNA was isolated from cultured THP-1 cells using TRIzol reagent (1 ml, Invitrogen). Total RNA sample (1 *μ*g) was reverse transcribed into complementary DNA (cDNA) using SuperScript III First-Strand Synthesis System (Invitrogen). The sequences of the primers used are shown in Table 1. DNA amplification was performed on a PCR thermal cycler (Thermo Fisher Scientific) using the following thermal conditions: an initial step at 95°C for 10 min, followed by 40 cycles of 95°C for 15 sec, 60°C for 1 min, and 95°C for 15 sec, and a final phase at 60°C for 5 min. The relative amount of mRNA of each sample was calculated using the 2^−ΔΔCT^ method and normalized by reference to the expression of *β*-actin (loading control).

### 2.14. Measurements of IL-6

The endometrial tissues were lysed, followed by centrifugation at 1500 rpm for 10 min. The supernatant was then collected and stored frozen at −80°C for further use. The concentrations of IL-6 in plasma and lysis supernatant were measured using an enzyme-linked immunosorbent assay (ELISA) kit (Thermo Fisher Scientific, Waltham, MA, USA) according to the manufacturer's instructions. The protein concentration in lysis supernatant was quantified with a BCA kit. Thus, the IL-6 concentration per mg of protein was calculated (pg/ml/mg protein). All assays were performed in triplicate in three independent experiments.

### 2.15. Statistical Analysis

The SPSS for Windows version 19.0 software package (SPSS Inc., Chicago, IL, USA) was used for statistical data analysis. Quantitative data are presented as the mean ± standard deviation (SD) from independent experiments in triplicate. Comparisons between two groups were performed using independent-samples *t*-tests or nonparametric Mann–Whitney *U*-test as appropriate. *P* < 0.05 was regarded as statistically significant.

## 3. Results

### 3.1. Macrophage Polarization in Peritoneal Fluid

**Figure 1:**
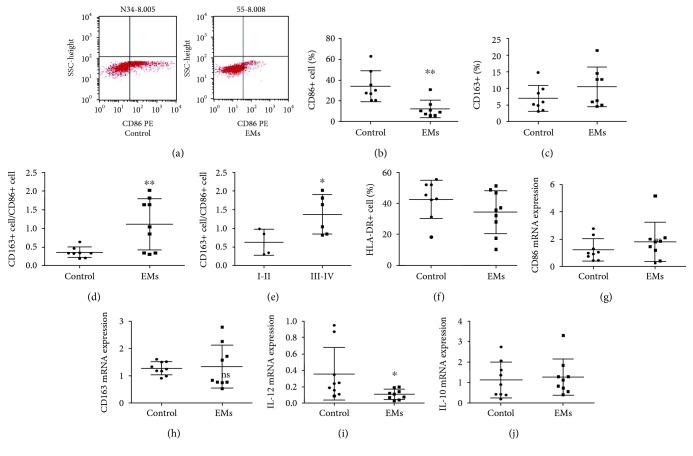
Cytokine concentrations and macrophage percentages in peritoneal wash solutions. (a) Flow cytometric analysis of peritoneal macrophages in control participants and endometriosis patients; (b) percentages of CD86+ macrophages; (c) percentages of CD163+ macrophages; (d) ratio of CD163+/CD86+ macrophages; (e) ratio of CD163+/CD86+ macrophages in endometriosis patients with stage I + II versus stage III + IV disease; (f) percentages of HLA-DR+ macrophages; (g) CD86 mRNA; (h) CD163 mRNA; (i) IL-12 mRNA; (j) IL-10 mRNA. EMs: endometriosis patients. *n* = 8–10, ^∗^ *P* < 0.05, ^∗∗^ *P* < 0.01.

Cytokine concentrations and macrophage percentages in peritoneal wash solutions were determined (Figure 1). The percentages of peritoneal macrophages from control participants that stained positively for CD86, HLA-DR, and CD163 were 21%–62%, 31–55%, and 3–14%, respectively. There were no significant differences in the percentages of HLA-DR+ or CD163+ macrophages between control participants and endometriosis patients. However, compared with that in control participants, the percentage of CD86+ macrophages in endometriosis patients was decreased. The ratio of CD163+/CD86+ macrophages was elevated in endometriosis patients, especially in patients with advanced endometriosis (stages III-IV), who had a significantly higher ratio of CD163+/CD86+ macrophages than those with stage I-II endometriosis.

In addition, the expression levels of CD163 mRNA and CD86 mRNA as well as cytokine IL-10 mRNA were not significantly different between the two groups, whereas the expression of IL-12 mRNA in the peritoneal macrophages of endometriosis patients was decreased, as compared with that in control samples.

### 3.2. Eutopic and Ectopic Endometrial Homogenates Induced Immunological Tolerance of THP-1 Cells

**Figure 2:**
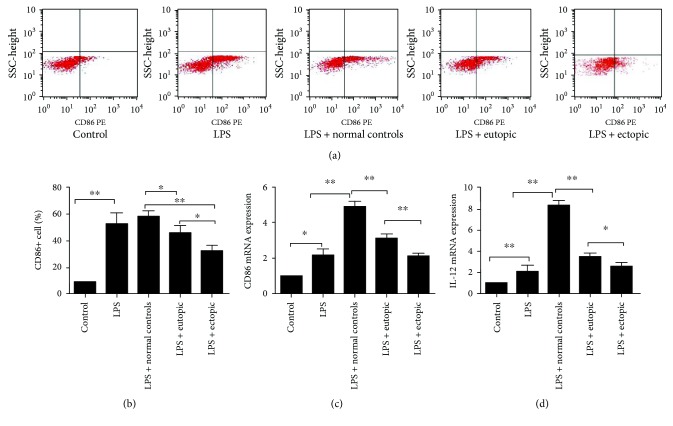
Eutopic and ectopic endometrial homogenates induced immunological tolerance of THP-1 cells. THP-1 cell-derived macrophages were treated with either eutopic or ectopic endometrial homogenate or normal endometrial homogenate for 72 h and then stimulated by LPS for 24 h. According to flow cytometric analysis (a) and RT-PCR, the percentage of CD86+ cells (b) as well as CD86 mRNA (c) and IL-12 mRNA (d) levels was significantly decreased in THP-1 cell-derived macrophages treated with eutopic or ectopic endometrial homogenate from endometriosis patients, as compared with their counterparts. LPS: lipopolysaccharide. *n* = 8–10, ^∗^ *P* < 0.05, ^∗∗^ *P* < 0.01.

THP-1 cells were induced to differentiate into macrophage-like cells by treatment with PMA for 3 days. The macrophages were treated with either eutopic or ectopic endometrial homogenate from endometriosis patients or endometrial homogenate from normal controls for 72 h. Upon stimulation by LPS for 24 h, the percentage of CD86+ cells as well as the CD86 mRNA and IL-12 mRNA levels was significantly decreased in THP-1 cell-derived macrophages treated with eutopic or ectopic endometrial homogenate from endometriosis patients, as compared with levels in those treated with normal endometrial homogenates (Figure 2).

### 3.3. Ectopic Endometrial Homogenates Induced M1 to M2 Polarization of Macrophages

**Figure 3:**
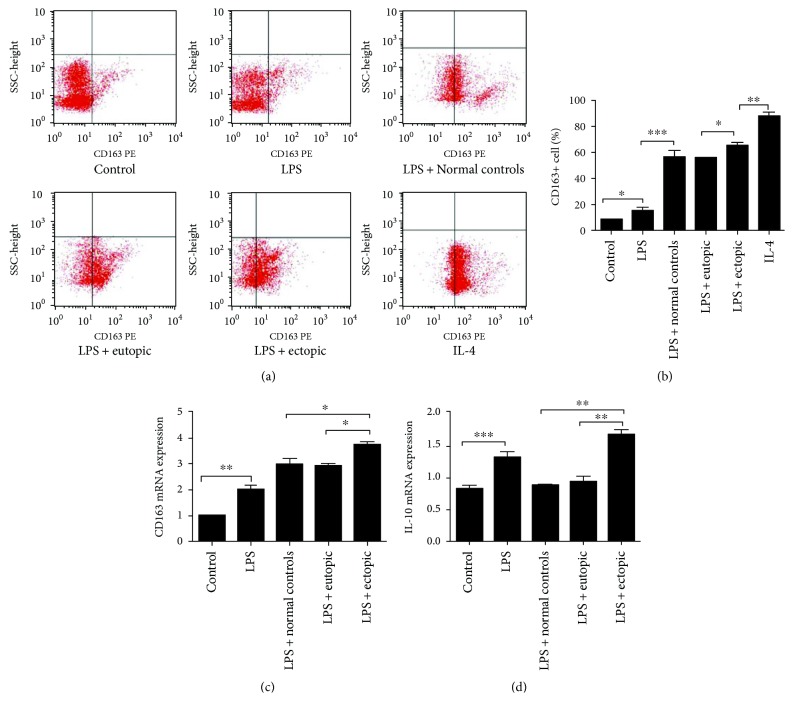
Ectopic endometrial homogenates induced M1 to M2 polarization of macrophages. THP-1 cell-derived macrophages were subjected to LPS stimulation for 24 h, followed by treatment with either eutopic or ectopic endometrial homogenate or normal endometrial homogenate for 72 h. As detected by flow cytometric analysis (a) and RT-PCR, the percentage of CD163+ cells (b) as well as CD163 mRNA (c) and IL-10 mRNA (d) levels was significantly decreased in THP-1 cell-derived macrophages treated with ectopic endometrial homogenate, as compared with normal endometrial homogenates. However, there were no significant differences in the percentage of CD163+ cells or CD163 mRNA and IL-10 mRNA levels between THP-1 cell-derived macrophages treated with eutopic and normal endometrial homogenate. LPS: lipopolysaccharide. *n* = 10, ^∗^ *P* < 0.05, ^∗∗^ *P* < 0.01.

THP-1 cell-derived macrophages were subjected to LPS stimulation for 24 h, followed by treatment with either eutopic or ectopic endometrial homogenate or normal endometrial homogenate for 72 h. There were no significant differences in the percentages of CD163+ cells or CD163 mRNA and IL-10 mRNA levels between THP-1 cell-derived macrophages treated with eutopic and normal endometrial homogenate. However, addition of ectopic endometrial homogenate to THP-1 cells induced an increased percentage of CD163+ cells as well as elevated CD163 mRNA and IL-10 mRNA levels, indicating the ability of ectopic endometrial homogenate to induce the polarization of M1 macrophages into the M2 phenotype (Figure 3).

### 3.4. Signaling Pathways Underlying the Macrophage Polarization by Ectopic Endometrial Homogenate

**Figure 4:**
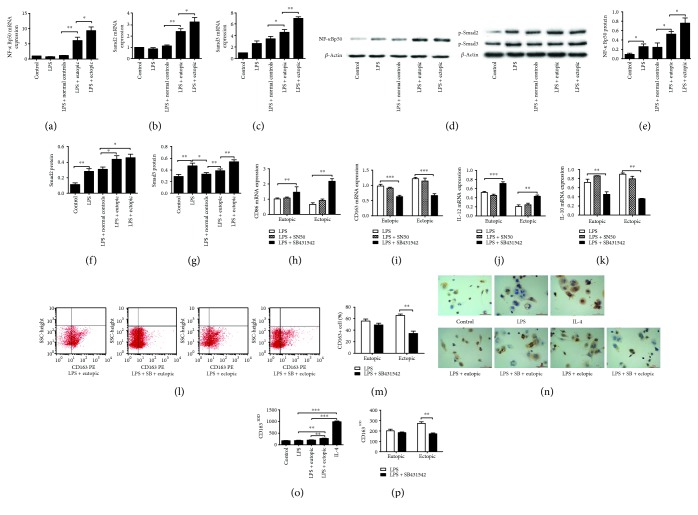
Signaling pathways underlying the macrophage polarization induced by ectopic endometrial homogenate. THP-1 cell-derived macrophages were treated with either eutopic or ectopic endometrial homogenate or normal endometrial homogenate for 72 h and then subjected to LPS stimulation for 24 h. The mRNA levels of NF-*κ*Bp50 (a), Smad2 (b), and Smad3 (c) were detected by RT-PCR. The band intensities (d) were used for quantitative analysis of NF-*κ*Bp50 (e), Smad2 (f), and Smad3 (g) protein levels on western blotting. THP-1 cell-derived macrophages were preincubated with NF-*κ*Bp50 inhibitor SN50 or Smad2/Smad3 inhibitor SB431542 before addition of homogenates. The mRNA levels of CD86 (h), CD163 (i), IL-12 (j), and IL-10 (k) were detected by RT-PCR. Flow cytometric analysis (l) showed that inhibition of Smad2/Smad3 with SB431542 resulted in a decreased percentage of CD163+ cells in the ectopic group, but not in the eutopic group, in response to LPS stimulation (m). Immunohistochemical staining analysis (n) showed that macrophages treated with ectopic endometrial homogenate had significantly higher IOD values for CD163 staining than those treated with eutopic (o). However, SB431542 could abolish this increasing trend in the ectopic group in the presence of LPS (p). LPS: lipopolysaccharide; SB: SB431542. *n* = 10, ^∗^ *P* < 0.05, ^∗∗^ *P* < 0.01.

THP-1 cell-derived macrophages were treated with either eutopic or ectopic endometrial homogenate or normal endometrial homogenate for 72 h and then subjected to LPS stimulation for 24 h. The macrophages treated with either eutopic and ectopic endometrial homogenate exhibited increased mRNA levels of NF-*κ*Bp50, Smad2, and Smad3, compared with control cells. In particular, the mRNA levels of these genes in the ectopic group were significantly higher than those in the eutopic group. Similarly, western blot analysis showed significantly higher protein expression of NF-*κ*Bp50, Smad2, and Smad3 in macrophages treated with eutopic or ectopic endometrial homogenate compared with levels in control samples (Figures 4(a)-4(g)).

For investigation of the role of NF-*κ*Bp50 and Smad2/Smad3 in the polarization of macrophages, THP-1 cell-derived macrophages were preincubated with the NF-*κ*Bp50 inhibitor SN50 for 1 h or the Smad2/Smad3 inhibitor SB431542 for 24 h. Preincubation with SB431542 resulted in decreased mRNA levels of CD163 and IL-10 and increased mRNA levels of CD86 and IL-12 in both the eutopic and ectopic groups. However, no similar results were observed in macrophages treated with SN50 (Figures 4(h)–4(k)). By flow cytometric analysis, inhibition of Smad2/Smad3 with SB431542 resulted in a decreased percentage of CD163+ cells in the ectopic group, but not in the eutopic group, in response to LPS stimulation. Immunohistochemical staining analysis showed that macrophages treated with ectopic endometrial homogenate had significantly higher IOD values for CD163 staining than those treated with eutopic or normal endometrial homogenate. However, SB431542 could abolish this increasing trend in the ectopic group in the presence of LPS.

### 3.5. Effect of Serum from Endometriosis Patients on Macrophage Polarization

**Figure 5:**
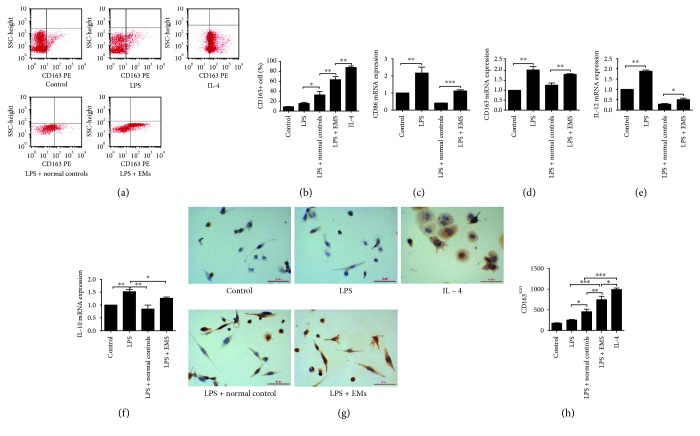
Effect of serum from endometriosis patients on macrophage polarization. Flow cytometric analysis (a) showed that treatment with serum from endometriosis patients resulted in an increased percentage of CD163+ macrophages in response to LPS stimulation as compared with the control (b). The mRNA levels of CD86 (c), CD163 (d), IL-12 (e), and IL-10 (f) were detected by RT-PCR. Immunohistochemical staining analysis (g) revealed significantly increased intensity of CD163 staining in macrophages treated with serum from endometriosis patients compared with that in controls (h). LPS: lipopolysaccharide. *n* = 12, ^∗^ *P* < 0.05, ^∗∗^ *P* < 0.01.

THP-1 cell-derived macrophages were subjected to LPS stimulation for 24 h, followed by treatment with serum from endometriosis patients or normal controls for 6 days. Compared with that in controls, the percentage of CD163+ macrophages in endometriosis patients increased in response to LPS stimulation (Figure 5). In addition, the mRNA levels of CD86 and CD163 as well as the cytokine IL-12 were significantly higher in macrophages treated with serum from endometriosis patients than in those treated with serum from normal controls. Similarly, immunohistochemical staining analysis showed a significantly increased intensity of CD163 staining in macrophages treated with serum from endometriosis patients compared with that in macrophages treated with serum from controls.

### 3.6. Signaling Pathways Underlying Macrophage Polarization in Response to Serum from Endometriosis Patients

**Figure 6:**
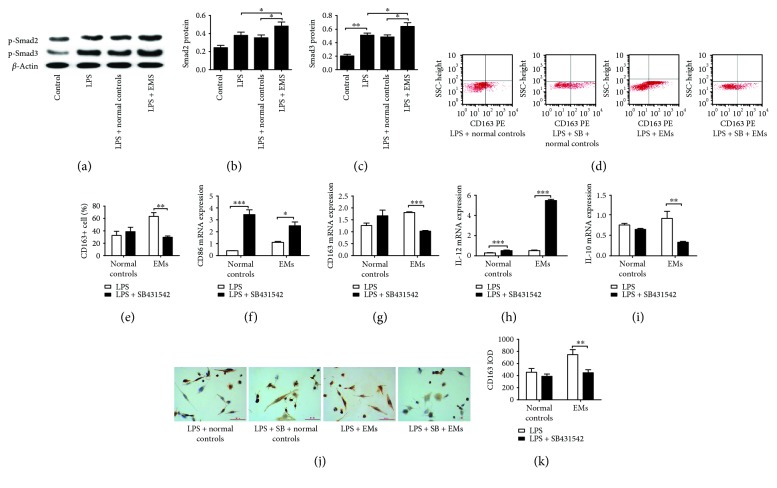
Signaling pathways underlying the macrophage polarization by serum from endometriosis patients. Western blot analysis (a) showed that the protein levels of Smad2 (b) and Smad3 (c) were increased in THP-1 cell-derived macrophages treated with serum from endometriosis patients in the presence of LPS, as compared with the control. By flow cytometric analysis (d), preincubation with SB431542 resulted in a decrease in the percentage of CD163+ cells, which was only observed in macrophages treated with serum from endometriosis patients (e). The mRNA levels of CD86 (f), CD163 (g), IL-12 (h), and IL-10 (i) were detected by RT-PCR. Immunohistochemical staining analysis (j) showed significantly decreased intensity of CD163+ macrophage staining in the presence of serum from endometriosis patients after preincubation with SB431542 (k). LPS: lipopolysaccharide; SB: SB431542. *n* = 12, ^∗^ *P* < 0.05, ^∗∗^ *P* < 0.01.

Western blot analysis showed that the protein expression of Smad2 and Smad3 was increased in THP-1 cell-derived macrophages treated with serum from endometriosis patients in the presence of LPS, as compared with that in control cells (Figure 6). Preincubation with the Smad2/Smad3 inhibitor SB431542 resulted in a decrease in the percentage of CD163+ cells, which was only observed in macrophages treated with serum from endometriosis patients. Similarly, after preincubation with SB43154, the mRNA expression levels of CD163 and IL-10 were decreased while CD86 and IL-12 expression was increased in macrophages treated with serum from endometriosis patients. Immunohistochemical staining analysis showed a significantly decreased intensity of CD163+ macrophage staining in the presence of serum from endometriosis patients after preincubation with SB431542.

### 3.7. Effect of IL-6 on Macrophage Polarization

**Figure 7:**
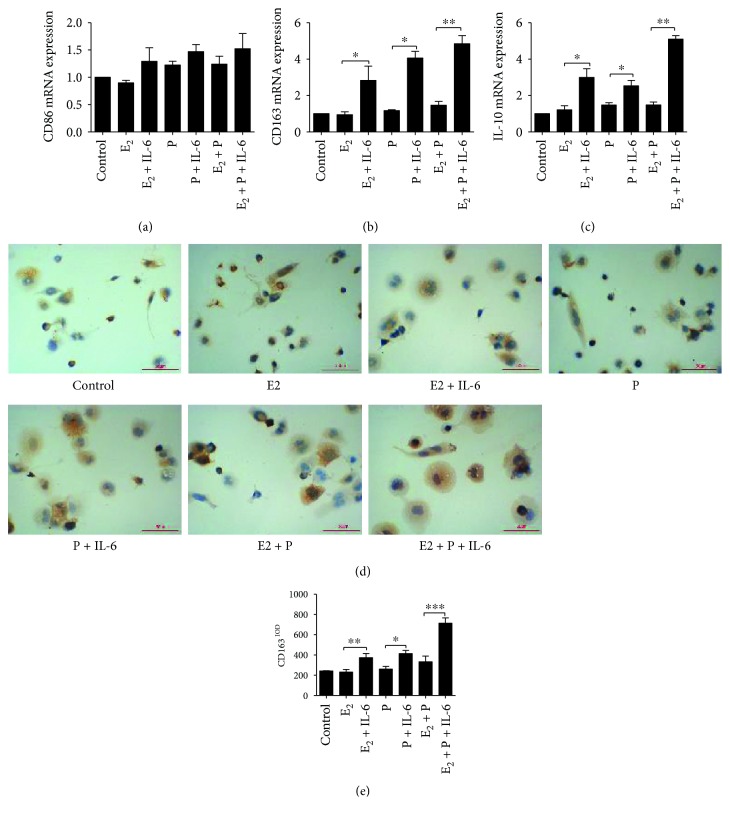
Effect of IL-6 on the polarization of macrophages. The mRNA expression levels of CD86 (a), CD163 (b), and IL-10 (c) were detected by RT-PCR. Immunohistochemical staining analysis (d) showed significantly increased intensity of CD163 staining after culture in IL-6-induced conditioned medium, but not estrogen- or progestin-induced conditioned medium (e). *n* = 8, ^∗^ *P* < 0.05, ^∗∗^ *P* < 0.01.

The expression levels of CD86, CD163, and IL-10 were detected in THP-1 cells after treatment with conditioned medium. The mRNA expression levels of CD163 and IL-10, but not CD86, were significantly increased after cells were cultured in IL-6-induced conditioned medium (Figure 7). In contrast, expression of these mRNAs remained largely unchanged in cells treated with estrogen and/or progestin-induced conditioned medium but without induction by IL-6. Moreover, immunohistochemical staining analysis showed a significantly increased intensity of CD163 staining after cells were cultured in IL-6-induced conditioned medium, but not after culture in estrogen- or progestin-induced conditioned medium. These data suggest that IL-6-induced conditioned medium produced by ESCs could promote the polarization of macrophages to the M2 phenotype.

**Figure 8:**
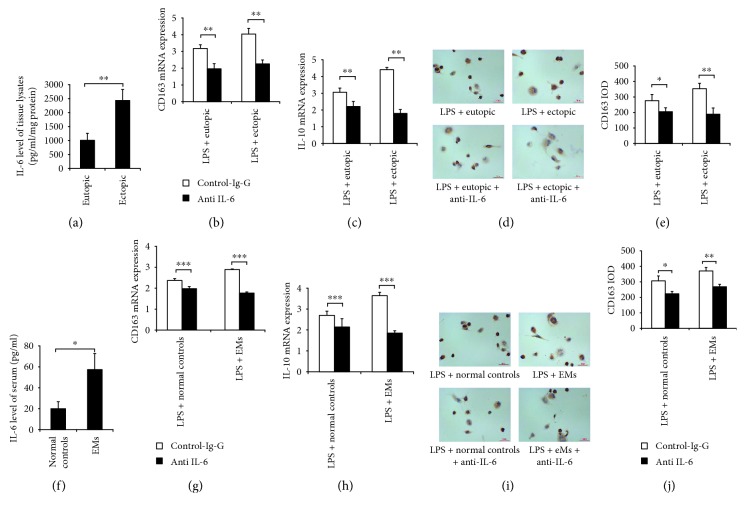
IL-6 neutralizing antibody assays. THP-1 cell-derived macrophages were sequentially treated with 100 ng/ml LPS for 24 h, 2 *μ*g/ml IL-6 neutralizing antibody (anti-IL-6), or IgG isotype control antibody (control-IgG) for 24 h and then cultured with endometrial homogenates or serum from endometriosis patients or control subjects. (a) IL-6 concentration in lysis supernatant of endometrial tissues. After treatment with 100 μl/ml eutopic or ectopic endometrial homogenate for 72 h, the mRNA expression levels of CD163 (b) and IL-10 (c) were detected by RT-PCR. Immunohistochemical staining (d) and quantitative analysis of CD163+ cells (e). (f) Serum IL-6 concentration. After treatment with 10% serum from control subjects versus endometriosis patients for 6 days, the mRNA expression levels of CD163 (g) and IL-10 (h) were detected by RT-PCR. Immunohistochemical staining (i) and quantitative analysis of CD163+ cells (j). LPS: lipopolysaccharide; EMs: endometriosis patients. *n* = 6, ^∗^ *P* < 0.05, ^∗∗^ *P* < 0.01, ^∗∗∗^ *P* < 0.001.

The IL-6 concentration in the lysis supernatant of ectopic endometrial homogenate was significantly higher than that in eutopic endometrial homogenate (Figure 8(a)). Upon blocking with IL-6 neutralizing antibody, THP-1 cell-derived macrophages exhibited decreased expression of CD163 and IL-10 after treatment with eutopic or ectopic endometrial homogenate, as shown by RT-PCR and immunohistochemical staining analyses (Figures 8(b)–8(e)). Similarly, a higher serum IL-6 concentration was found in patients with endometriosis than in control participants (Figure 8(f)), and macrophages showed decreased expression of CD163 and IL-10 after blockage of IL-6 (Figures 8(g)–8(j)).

## 4. Discussion

In this study, we found a decrease in the percentage of CD86+ macrophages and an increase in the ratio of CD163+/CD86+ macrophages in peritoneal washings of endometriosis patients, especially those with advanced disease, indicating that retrograde menstruation into the abdominal cavity could induce immune tolerance of peritoneal macrophages. Ectopic endometrial homogenate could promote M1 to M2 macrophage polarization, as evidenced by an increased percentage of CD163+ macrophages and elevated IL-10 expression as well as a decreased percentage of CD86+ cells and lower expression of IL-12, probably via the TGF-*β*-SMAD2/3 pathway. Interestingly, serum of women with endometriosis seemed to induce the polarization of macrophages towards both M1 and M2 phenotypes. IL-6 facilitated macrophage M2 polarization in endometriosis, and neutralization of IL-6 diminished this effect. Thus, this study provides detailed evidence supporting alterations in M1 to M2 macrophage polarization that may contribute to the initiation as well as progression of endometriosis.

The M1/M2 dichotomy of macrophage polarization has been implicated in the susceptibility/resistance to endometriosis, but with inconsistent opinions. In this study, as compared with control samples, there was a decrease in M1 macrophages in peritoneal wash solutions, as evidenced by a decreased percentage of CD86+ macrophages as well as downregulation of the IL-12 mRNA level in macrophages, whereas the percentage of CD163+ macrophages remained unchanged. The reduction in M1 macrophages, which have a role in fighting invading pathogens, may facilitate survival of shed endometrial tissue and invasion of the mesothelial layer. A previous study by Itoh et al. showed that endometriosis patients exhibited a significantly higher number of macrophages but a similar ratio of CD163+ M2 cells in the peritoneal fluid in comparison with controls [21]. Bacci et al. found that peritoneal macrophages from endometriotic patients and control participants similarly expressed the CD86 receptor [16]. The discrepancies among studies may have been caused by examining various macrophage subpopulations using different detection methods. On the other hand, macrophages are heterogeneous cells exhibiting great diversity in morphology, transcriptional profiles, anatomical distribution, and functional capabilities. The researchers may have detected cells at different time points during the process by which macrophages undergo dynamic transitions between different functional states.

In this study, we found that compared with normal control samples, both eutopic and ectopic endometrial homogenates could significantly decrease the percentage of CD86+ macrophages and downregulate mRNA levels of CD86 and IL-12 in macrophages in response to LPS stimulation, suggesting that both homogenates could induce immune tolerance and inhibition of proinflammatory factors. On the other hand, ectopic endometrial homogenate could increase the percentage of CD163+ macrophages as well as upregulation of mRNA levels of CD163 and IL-10 in the presence of LPS intervention, indicating a transition from M1 to M2 phenotype. Our results were consistent with the findings of Mei et al., who reported that when cocultured with ectopic ESCs, macrophages expressed higher levels of CD163 and CD209 but lower levels of HLA-DR and CD11c, accompanied by increased secretion of IL-10 and decreased secretion of IL-12 [22]. Macrophage M2 polarization has been found to promote the angiogenesis in ectopic endometrium, facilitate proliferation of ESCs, and favor the growth and invasion of endometriotic lesions [23]. Therefore, we hypothesize that macrophages, which are recruited to the peritoneal cavity during the process of retrograde menstruation, induce immune tolerance to favor the ectopic implantation of the endometrium, and then successfully implanted ectopic endometrium secretes various cytokines such as IL-6, IL-8, and monocyte chemoattractant protein-1, which further recruit macrophages and facilitate M2 polarization to promote the growth of ectopic lesions [3, 24].

Dynamic interplay among cytokines may favor the realization of a permissive microenvironment for the implantation and growth of ectopic endometrium. Previous studies showed that serum of women with endometriosis had higher concentrations of IL-10, TNF-*α*, and TGF-*β*, and administration of these cytokines, such as IL-10, promoted the growth of endometriotic lesions in a murine model [20, 24]. Monocyte-derived dendritic cells that were conditioned with serum from women with endometriosis displayed a tolerogenic phenotype, including downregulation of CD86 and HLA-DR, as well as increased IL-10 but decreased IL-12 production [25]. The question is whether the serum of women with endometriosis exerts an impact on macrophage polarization. In our study, we found that the serum of patients with endometriosis could induce higher percentage of CD163+ macrophages and upregulate mRNA levels of CD86, CD163, and IL-12. These data indicate the polarization of macrophage towards both M1 and M2 phenotypes to maintain the balance between M1 and M2 macrophages that is of great significance to protecting tissues and organs. Chong et al. [26] reported a subset of CD163+ macrophages that displayed mixed polarizations, as evidenced by coexpression of M1 (CXCL10, TNF-*α*, and CD127) and M2 (CD209 and TGF-*β*) macrophage-related proteins in discoid lupus skin. Given that endometriosis is an autoimmune disease, we speculate that the macrophages treated with serum of patients with endometriosis may simultaneously express CD86 and CD163, representing a special type of M2 macrophages. Confirming these findings with certainty, however, will require further investigation.

In this study, we observed upregulation of Smad2/Smad3 in macrophages after treatment with eutopic and ectopic endometrial homogenates or serum of women with endometriosis in response to LPS stimulation, whereas blockage of Smad2/Smad3 with their inhibitor SB431542 could reverse the macrophage polarization from M1 to M2, suggesting the involvement of the TGF-*β*-SMAD2/3 signal transduction pathway in the M1 to M2 polarization induced by both ectopic endometrial homogenate and serum of women with endometriosis. In addition, an increase in NF-*κ*Bp50 was also observed after intervention. However, the NF-*κ*Bp50 inhibitor SN50 seemed to fail to prevent M1 to M2 polarization, but we could not exclude the possible procedural factors, such as selection of an inhibitor with low specificity to NF-*κ*Bp50, insufficient dose, and insufficient time duration. Whether NF-*κ*Bp50 contributes to the development of endometriosis needs further exploration.

Previous studies have demonstrated that ectopic ESCs secrete more IL-6 than normal ESCs do [27–29], which is consistent with our findings. In this study, we prepared IL-6-induced conditioned medium by treating ESCs with or without various concentrations of estrogen or progestin. Conditioned medium induced by IL-6, but not estrogen or progestin, could facilitate M2 polarization. Burns et al. [30] using an estrogen-receptor *α* (ERα) knockout mice model discovered two phases of endometriosis, namely, a hormone-dependent phase and an immune-dependent phase. E_2_/ER*α*/IL-6-mediated cross talk played a partial role in the early initiation of endometriosis, whereas the IL-6-mediated response was found in developing lesions lacking ER*α*. On the other hand, neutralization of IL-6 with antibody decreased the expression of CD163 and IL-10 in macrophages in an endometriosis condition, reflecting prevention of macrophage polarization toward an M2 phenotype. Taken together, these findings indicate that IL-6 may mediate important biological events that contribute to the initiation as well as progression of endometriosis. These data will inspire new therapeutic strategies to treat endometriosis.

In conclusion, this study provides detailed evidence explaining the alterations in M1 to M2 macrophage polarization in endometriosis patients, which might contribute to the initiation as well as progression of endometriosis. Further investigations are necessary to improve our understanding of the pathological changes in endometriosis.

## Figures and Tables

**Table 1:** Primers used for quantitative RT-PCR analysis.

Gene name	Primers
CD86	F: 5′-TCATTCCCTGATGTTACGAG-3′ R: 5′-GCCGCTTCTTCTTCTTCC-3′
CD163	F: 5′-TGGACCGATATGGCTCAATG-3′ R: 5′-AGCGACCTGTTGTGGCTTTT-3′
IL-10	F: 5′-TGAGAACCAAGACCCAGACATCA-3′ R: 5′-GGCATTCTTCACCTGCTCCAC-3′
IL-12p40	F: 5′-GCGGAGCTGCTACACTCTC-3′ R: 5′-CCATGACCTCAATGGGCAGAC
*β*-Actin	F: 5′-ACCCTGAAGTACCCCATCGAG-3′ R: 5′-AGCACAGCCTGGATAGCAAC-3′
NF-*κ*Bp50	F: 5′-AATAGCCTGCCATGTTTGCT-3′ R: 5′-TGCCAATGAGATGTTGTCGT-3′
Smad2	F: 5′-ATGTCGTCCATCTTGCCATT-3′ R: 5′-TTTTCTTCCTGCCCATTCTG-3′
Smad3	F: 5′-TGCTGAGACTGACCCAAGTG-3′ R: 5′-AAATTGGGAAGAAGCGAGGT-3′

## Data Availability

The data used to support the findings of this study are available from the corresponding author upon request.

## Supplementary Materials

Supplementary Materials Figure 1: flow chart of the study.
