# Supplementary figures and images for: Serum and Ectopic Endometrium from Women with Endometriosis Modulate Macrophage M1/M2 Polarization via the Smad2/Smad3 Pathway
Source: J Immunol Res. 2018 Sep 12;2018:6285813. doi: 10.1155/2018/6285813 (PMC6157144; doi:10.1155/2018/6285813)

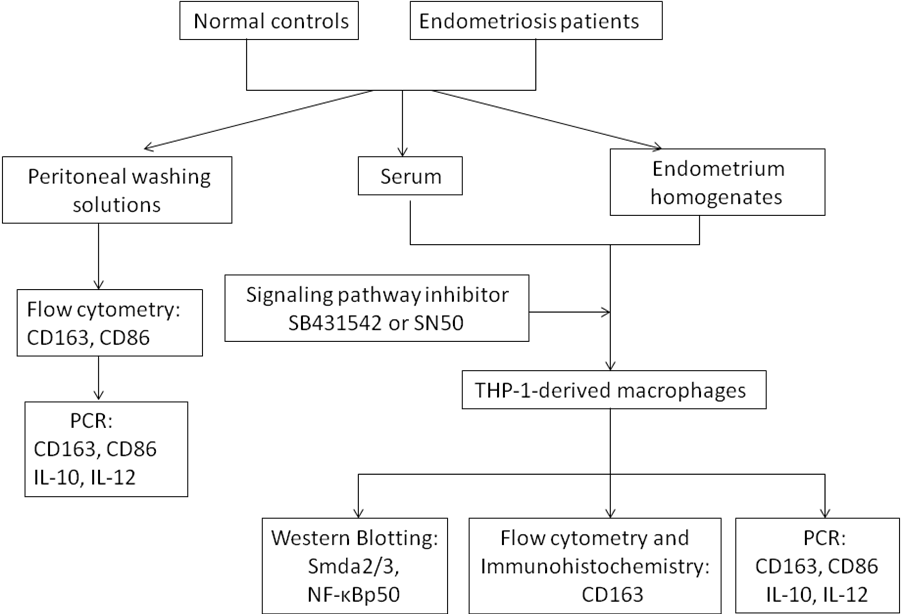


**Supplementary Fig. 1 Flow chart of the study.**

Supplement: Supplementary Materials — Figure 1: flow chart of the study. [file 6285813.f1.doc]
